# Investigation on X-ray Photocurrent Response of CdZnTe Photon Counting Detectors

**DOI:** 10.3390/s20020383

**Published:** 2020-01-09

**Authors:** Yingrui Li, Gangqiang Zha, Yu Guo, Shouzhi Xi, Lingyan Xu, Wanqi Jie

**Affiliations:** 1 State Key Laboratory of Solidification Processing, and MIIT Key Laboratory of Radiation Detection Materials and Devices, Northwestern Polytechnical University, Xi’an 710072, China; li_yrui@foxmail.com (Y.L.); guoyu_@mail.nwpu.edu.cn (Y.G.); xiaola507@163.com (S.X.); xulingyan@nwpu.edu.cn (L.X.); jwq@nwpu.edu.cn (W.J.); 2 Imdetek Co., Ltd., Shaanxi Xixian New Area Qinhan New City Tian Gong 1 road 8-1, Xi’an 712000, China

**Keywords:** CdZnTe, X-ray photocurrent, photon counting, count rate, critical flux, photoconductive gain

## Abstract

Counting rate is an important factor for CdZnTe photon counting detectors as high-flux devices. Until recently, there has been a lack of knowledge on the relationship between X-ray photocurrent response and the photon counting performance of CdZnTe detectors. In this paper, the performance of linear array 1 × 16-pixel CdZnTe photon counting detectors operated under different applied biases is investigated. The relation between experimental critical flux and applied bias show an approximate quadratic dependence, which agrees well the theoretical prediction. The underlying relationship among X-ray photocurrents, carrier transport properties, and photon counting performance was obtained by analyzing X-ray current–voltage and time current curves. The typical X-ray photocurrent curve can be divided into three regions, which may be explained by the photoconductive gain mechanism and electric field distortion characteristics. To keep CdZnTe photon counting detectors working in a “non-polarized state”, the applied bias should be set on the left side of the “valley region” (high bias direction) in the X-ray I-V curves. This provides an effective measurement for determining the proper working bias of CdZnTe detectors and screening photon counting detector crystals.

## 1. Introduction

Detectors based on direct converting semiconductors have been used for the detection of X-ray and gamma rays for more than a decade [1,2]. Due to a wide bandgap of 1.5 eV at 300 K, excellent electron transport properties, high density, and low leakage current, CdZnTe is currently considered a suitable candidate for high-efficiency, energy-resolved X-ray photon counting applications, including computed industrial gauging, transportation security, medical and industrial imaging, and others [3,4,5,6,7]. There is a growing interest in the potentials of pulse mode CdZnTe detector technology for high-flux, multi-energy binning imaging. Applications such as medical imaging (computed tomography), need the capability to detect a photon flux in the order of hundred million mm^−2^s^−1^, or higher [8,9]. Such a detector is still a challenge because of the “polarization” effect [10,11,12]. The polarization effect is closely related to hole transport performance [13]. At high incident X-ray flux, the build-up of space charge formed by trapped holes within the crystals produces an internal electric field which acts to oppose the externally applied voltage. Eventually, the effective field across the device is significantly reduced, and even complete collapsed in extreme conditions. Such polarization phenomena can cause a significant reduction in signal amplitude, hence the signal amplitude falls below the detection threshold which results in catastrophic device failure [14]. Therefore, a clear understanding of the charge transport properties of CdZnTe photon counting detectors, and determining their operating conditions (i.e., applied voltage), is essential for future high-flux applications. Most experiments and theoretical calculations were done focusing on the study of electric field distortion across the device and its effect on the carrier collection efficiency under high flux conditions [13,15,16]. However, there has been less research on the coupling relationship between X-ray photocurrent response and the photon counting performance of CdZnTe detectors.

In this work, we explored the influence of intense X-ray irradiation on the performance of linear array 1 × 16-pixel CdZnTe photon counting detectors operated in different applied biases. The focus of the experiments was on the X-ray photocurrent response of the detector under different flux conditions. The underlying relationship among photon counting performance, X-ray photocurrent response, and carrier transport properties of CdZnTe detectors under high flux X-ray irradiation was analyzed. Based on these results, a simple method to evaluate the minimum required applied bias of CdZnTe detectors under a certain X-ray dose was proposed, without bonding them to the complicated application-specific integrated circuit (ASIC) readout system. This provided an effective method for the screening and grading of CdZnTe photon counting detector crystals and the determination of detector operating conditions.

## 2. Experimental Section

**Figure 1:**
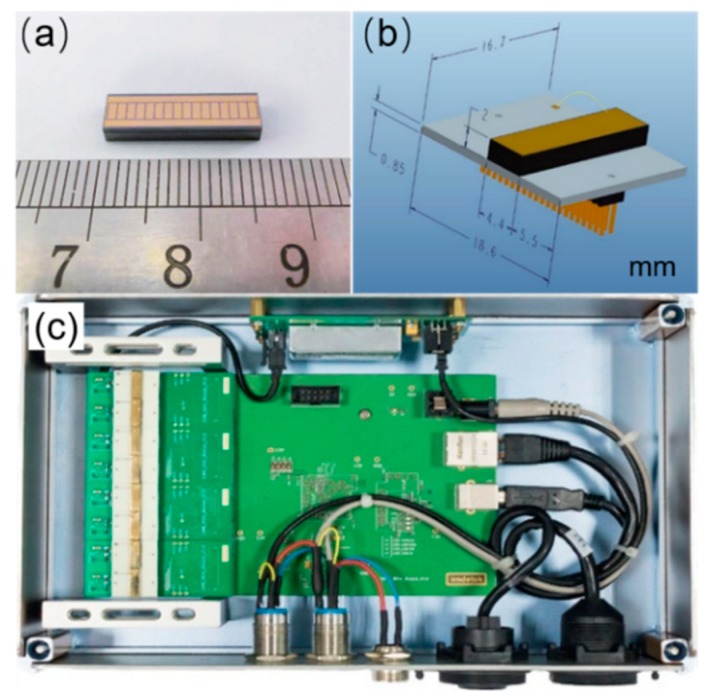
(**a**) Anode structure of linear array 1 × 16-pixel CdZnTe detectors; (**b**) packaging structure of CdZnTe photon counting detectors; (**c**) MXA-L256P readout system equipped with 8 CdZnTe photon counting detectors.

The linear array 1 × 16-pixel photon counting detectors were fabricated from 16.6 mm × 4.4 mm × 2 mm CdZnTe single crystals grown by the modified vertical Bridgman method from Imdetek Ltd. The mobility-lifetime products of electrons and holes in the CdZnTe detector were 1.90 × 10^−3^ cm^2^V^−1^ and 3.73 × 10^−5^ cm^2^V^−1^ respectively, obtained by fitting the γ-ray energy spectra under different bias voltages using the Hetch equation. The device structure is shown in Figure 1a,b. The anode of the CdZnTe detector was fabricated with 0.9 mm × 1.8 mm area pixels forming a 1 × 16 array with a 0.1 mm pitch. The charge sharing effect can be ignored due to the suitable electrode structure [17]. The pixel array was surrounded by a guard electrode to eliminate possible side-surface leakage current and electric-field distortion effects. The detector cathode was prepared with a planar electrode. All electrodes were deposited by vacuum evaporation with Au. The CdZnTe detector pixel anodes were assembled 1:1 on dedicated printed circuit boards that enabled reliable probe testing of the device and facilitated the bonding of the device to a readout chip. The photon counting rate was obtained through a 16-channel application-specific integrated circuit (ASIC) readout system known as the MXA-L256P, developed by Imdetek Ltd., as shown in Figure 1c. This ASIC readout system was sufficiently fast and accurate to enable the processing of 10^6^ signals without significant count-rate degradation caused by pile-up effects, which means that pulse signals with a time interval greater than 1 μs could be resolved. Counting rate data were recorded with a detection threshold of 20 (~30 keV) to eliminate the interference of electronics noise on experimental results. The system temperature was controlled at 32 °C using a Hp-1320p thermostat. The measurement system contained an X-ray source (Spellman XRB011), and its tube current could be adjusted from 0 to 0.6 mA at a selected fixed tube voltage ranging from 40 to 80 kV. The X-ray dose increased linearly with the increasing tube current of the X-ray source. The distance of detectors and the X-ray source was 45 mm. The dose rate and tube current satisfied the conversion formula of y = 710.2× in this study and x, y represent the tube current (mA) and dose rate (μGy/s). The X-ray beam was collimated to the cathode area of the detectors and the detectors were side shielded to prevent scatter events. A Keithley 6517b electrometer was used to measure X-ray photocurrent I-V and I-t curves.

## 3. Results and Discussion

### 3.1. X-ray Photon Counting Response

**Figure 2:**
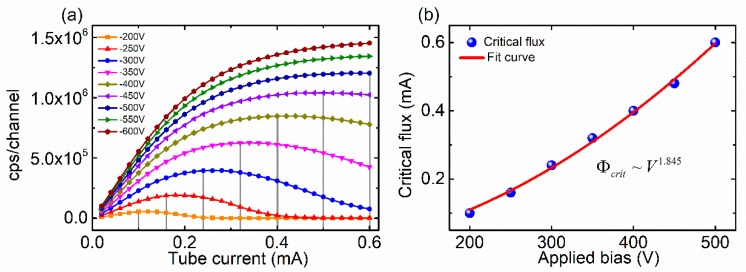
(**a**) Count rate performance of a typical pixel as a function of applied bias; (**b**) fit result of critical flux vs. applied bias.

Over 100 CdZnTe photon counting detectors were tested for the experimental study. The X-ray tube voltage was set to 80 kV and the tube current varied from 0 mA to 0.6 mA. Figure 2a shows typical results of the counting response to increasing X-ray flux for nine different bias voltages. At a relatively low bias voltage (below 450 V), as the X-ray dose gradually increases, the count rate curve first increases linearly and then deviates from linearity. When the incident dose increases to a certain value (critical flux), the count rate begins to decrease. It is estimated that the rise time of the pulse signal is about 200 ns at 200 V bias when the thickness of the detectors is 2 mm, and the electron mobility is considered to be 1000 cm^2^V^−1^s^−1^. The value of the rise time of the pulse signal was far less than 1 μs, which indicated that the decrease in the counting rate was not caused by the dead time of the detector. The reduction of count rate is due to detector polarization. This indicated that the trapping of charge carriers generated by X-ray photons, which formed a space charge region in the CdZnTe crystals, resulted in the deterioration of photon counting performance. 

At high bias (above 450 V), the count rate increases linearly and then gradually approaches saturation value, which is mainly limited by the pulse pile-up effect and the electronics dead time of the readout system. These results indicate that increasing the applied bias of CdZnTe detectors can improve the carrier collection efficiency (CCE), thereby increasing the pulse signal amplitude. When the pulse signal amplitude is higher than the signal threshold of the readout electronics, the detector count rate increases. This is shown in Figure 2a as an increase in the slope of the linear region of the count rate curve with increasing applied bias.

To further illustrate the relationship between applied bias and count rate response, the critical flux was measured as a function of the applied bias. Figure 2b shows the fit results of experimental critical flux vs. the applied voltage in an approximate quadratic dependence (b ≈ 1.845), which is in good agreement with Derek Bale’s theoretical predictions [16].

(1) $$\Phi_{crit}^{}\sim \frac{A\varepsilon V^{2}}{qL{\left(\lambda \beta \right)}^{2}}\left(\frac{\mu_{h}\tau_{h}}{\tau_{h}+\tau_{dtrap}}\right)$$

(2) $$\beta =1-e^{-L/\lambda }$$

where, the parameter *V* is the applied bias, *τ_dtrap_* is residence time of holes in hole traps, *μ_h_τ_h_* represents mobility-lifetime product of holes, *A, L, ε, q* are the detector area, thickness, the dielectric constant, and the elementary charge, respectively. *λ* is the characteristic length scale where a photon travels before interacting with the CdZnTe detectors. Increasing the detectors’ applied bias can improve the critical flux and change the detector from a “polarized state” to a “non-polarized state”. This indicates that the charge generated by X-ray irradiation is removed from the detector by drift and recombination at a sufficiently high rate. However, excessive applied bias voltage can lead to large leakage current, which places a burden on the ASIC readout electronics [18]. Based on the above analysis, CdZnTe photon counting detectors should work in a proper bias range and there is a minimum required bias voltage to keep the detectors operating in the “non-polarized state”.

### 3.2. X-ray Photocurrent Response

**Figure 3:**
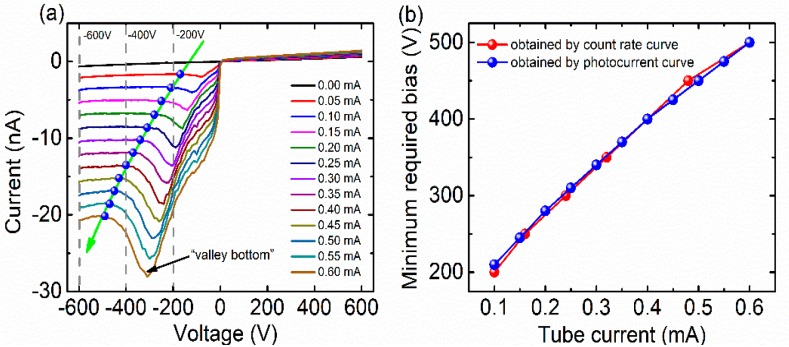
(**a**) I-V curve of a typical pixel at different tube current; (**b**) Minimum required bias voltage data obtained by count rate curve (see Figure 2a) and by X-ray photocurrent curve (**a**).

In order to study the relationship among photon counting performance, applied bias, and carrier transport properties of CdZnTe detectors under high flux X-ray irradiation, I-V curves were measured at different tube currents, as shown in Figure 3a. The X-ray tube voltage was set to 80 kV and the tube current varied from 0 mA to 0.6 mA. A “valley region” was observed in the X-ray photocurrent curve at all tube currents—the “valley region” was closely related to carrier transport properties under high flux irradiation. In the “valley region” bias range, the X-ray photocurrent first increases rapidly with the increase of applied bias voltage—after the “valley bottom” the photocurrent appears to decrease. The position of the “valley region” is closely related to the dose of X-ray radiation. As the tube current increases, the position of the “valley region” moves towards the high bias direction. The data point marked by the arrow in Figure 3a corresponds to the approximately lowest bias voltage on the left side of the “valley region”. Compared with the count rate curves shown in Figure 2a, we conclude that the applied bias should be set on the left of the “valley region”, i.e., the high bias direction to keep the detectors operating in a “non-polarized state”. Figure 3b shows the minimum required bias voltage for the CdZnTe detector at different X-ray doses. The bias voltage data obtained from Figure 3a fit well with the data obtained from the count rate curve (see Figure 2b). Based on the above analysis, we could determine the minimum required bias for CdZnTe detectors under a certain X-ray dose, and evaluate the critical flux when CdZnTe detectors operate at a certain applied bias by simply testing the X-ray I-V curve.

**Figure 4:**
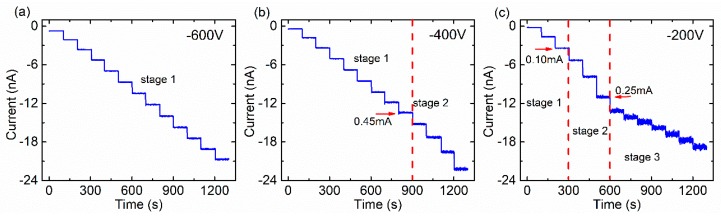
The X-ray photocurrent vs. time at the: (**a**) −600 V, (**b**) −400 V, (**c**) −200 V applied bias. The tube current varied from 0 mA to 0.6 mA, stepping 0.05 mA per 100 s. The X-ray tube voltage was set at 80 kV.

In order to gain understanding of the mechanism of the “valley region”, the X-ray I-t curves were measured at three typical bias voltages (−600 V, −400 V, and −200 V), as shown in Figure 4.

At −600 V bias, the X-ray I-t curve exhibits uniform steps when changing the X-ray flux, and the photocurrent remains stable at any tube current, as shown in Figure 4a. The contribution of the hole drift to photocurrent is negligible due to the short drift distance and the low mobility-lifetime product. In the simplest acceptable model, the photo-generated electrons are assumed to be homogeneously distributed in the detectors and the lifetime of photo-generated electrons is long, so the effect of capture and recombination can be ignored. The X-ray photons are almost completely absorbed at the cathode of the detector. The X-ray photocurrent can be described by both Formula (3) and Formula (4).

(3) $$I=\Delta nq\mu_{e}ES$$

(4) $$\Delta n=\;\;\;\overset{i=n}{\underset{1}{\sum }}\left[h\;\upsilon_{i}\right]\;\frac{\eta t}{E_{pair}}$$

where Δn is photo-generated electron density at steady state, and where *q*, $\mu_{e}$, *E*, *S* are electron charge, electron mobility, effective electric field strength, and electrode area respectively. h , υ are Planck constant and frequency. $\overset{i=n}{\underset{1}{\sum }}\left[h\;\upsilon^{i}\right]$ represents the total X-ray energy deposited to the detector per unit time. The η, *t*, $E_{pair}$ are quantum efficiency, transit time of photo-generated electrons, and the energy required to form an electron-hole pair, respectively. I is proportional to Δn and Δn is proportional to $\overset{i=n}{\underset{1}{\sum }}\left[h\;\upsilon^{i}\right]$, which indicates that the photocurrent increases in proportion to the increase of the tube current when the tube voltage is fixed.

The X-ray I-t curve at −400 V applied bias can be divided into two stages, as shown in Figure 4b. The I-t curve exhibits uniform steps in stage 1 similar to Figure 4a. In stage 2, a non-linear increase of the height of the photocurrent step was observed when the tube current exceeded 0.45 mA and the detector began to shift to a “polarized state”. This phenomenon may be explained by a so-called photoconductive gain mechanism [19,20]. When relatively low bias voltage (−400 V) was applied on the detector, the photo-generated holes can be easily captured by deep level traps near the cathode due to their poor mobility-lifetime product. With an increase in the X-ray dose, the positive space charges formed by the captured holes can be sufficient in density to reduce the width and the height of the Schottky barrier at the cathode. Thus, the electric field near the cathode is much higher than that across the detector [21]. In this case, thermal electrons immediately move across the barrier to satisfy charge conservation and constitute current, which indicates that the increasing injected electrons in the detector results in photocurrent gain. This effect may occur through field-dependent tunneling, thermionic emission, or a combination of both.

At −200 V bias, the X-ray I-t curve can be divided into three stages, as shown in Figure 4c. Stage 1 and stage 2 are similar to Figure 4b. In stage 3, a non-linear decrease of the photocurrent step is observed when the tube current changes to 0.25 mA. The X-ray I-t curve exhibits uniform steps as the tube current continues to increase. The height of the photocurrent step is lower than that in stage 1 in Figure 4c. Electric field distortion may be the main cause of this phenomenon. At a lower bias voltage, the rate of electron–hole generation by the X-ray photons can exceed the removal rate of charge by drift and recombination. The space charge formed by the trapped holes near the cathode can distort the electric field, thus the effective electric field strength of the detector decreases. This leads to a reduction in the height of the photocurrent step, even if a photoconductive gain mechanism exists. As the X-ray dose increases, the concentration of trapped holes near the cathode tends to be saturated, and the effective electric field strength of the detector gradually approaches a stable value that is responsible for the uniform photocurrent steps in stage 3. An obvious shake occurs in the photocurrent curve when the tube current exceeds 0.25 mA. This may be due to the rapid generation and recombination of photo-generated electrons and holes under distorted electric field conditions.

**Figure 5:**
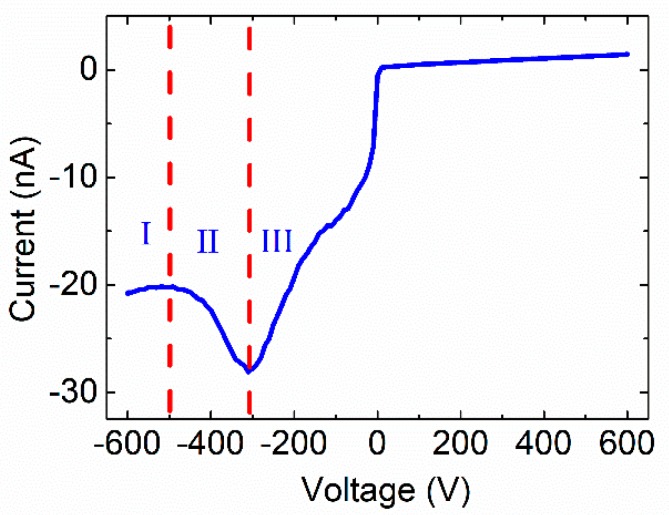
The typical X-ray photocurrent response of CdZnTe photon counting detectors. The tube current is 0.6 mA, the X-ray tube voltage was set to 80 kV.

The typical X-ray I-V curve can be simply divided into three regions, as shown in Figure 5. In region I, on the relatively high bias, the X-ray I-V curve follows Formula (3) and the detector operates in a “non-polarized” state. In region II, the X-ray I-V curve is dominated by the photoconductive gain process—the detector gradually changes to the “polarized state” as the applied bias decreases and the count rate of the detector gradually reduces. In region III, the trapped holes near the cathode tend to be saturated at low bias. The detectors tend to be totally polarized due to severe distortion of the electric field and the count rate is almost zero. Based on the above analysis, the working state of the CdZnTe photon counting detector can be judged by analyzing the shape of the X-ray I-V curve.

## 4. Conclusions

The photon counting properties of linear array 1 × 16-pixel CdZnTe detectors operated at different applied bias were studied. The experimental critical flux vs. applied voltage is in an approximate quadratic dependence (b ≈ 1.845). The X-ray photocurrent response characteristics of CdZnTe detectors are closely related to photon counting performance. The working state of CdZnTe photon counting detectors can be determined by analyzing X-ray I-V curves. The typical X-ray I-V curve can be divided into three regions according to the carrier transport characteristics. In region I, the CdZnTe photon counting detector works in a “non-polarized state” and the X-ray I-V curve can be described by Formula (3). In region II, the detector gradually polarized and the count rate began to decrease as the applied bias decreased. The X-ray photocurrent could be explained by the photoconductive gain mechanism. In region III, the detector tended to be totally polarized due to a severe distortion of the electric field, and the count rate was almost zero. The critical flux could be improved and the detector transformed from a “polarized state” to a “non-polarized state” by increasing the applied bias, and the applied bias could be set in the range of region I of the X-ray I-V curve to keep CdZnTe photon counting detectors working in the “non-polarized state”.

## References

[1] Wolf M.A., Umbarger C.J., Entine G. (1979). Use of a cadmium telluride detector in a new tiny personal radiation chirper. IEEE Trans. Nucl. Sci..

[2] Takahashi T., Watanabe S. (2001). Recent progress in CdTe and CdZnTe detectors. IEEE Transa. Nucl. Sci..

[3] Cajipe V.B., Calderwood R.F., Clajus M., Hayakawa S., Jayaraman R., Tumer T., Grattan B., Yossifor O. Multi-energy X-ray imaging with linear CZT pixel arrays and integrated electronics. Proceedings of the IEEE Symposium Conference Record Nuclear Science 2004.

[4] Schlomka J., Roessl E., Dorscheid R., Dill S., Martens G., Istel T., Bäumer C., Herrmann C., Steadman R., Zeitler G. (2008). Experimental feasibility of multi-energy photon-counting K-edge imaging in pre-clinical computed tomography. Phys. Med. Biol..

[5] Shikhaliev P.M. (2008). Energy-resolved computed tomography: First experimental results. Phys. Med. Biol..

[6] Szeles C., Cameron S.E., Soldner S.A., Ndap J.-O., Reed M.D. (2004). Development of the high-pressure electro-dynamic gradient crystal-growth technology for semi-insulating CdZnTe growth for radiation detector applications. J. Electron. Mater..

[7] Taguchi K., Iwanczyk J.S. (2013). Vision 20/20: Single photon counting x-ray detectors in medical imaging. Med. Phys..

[8] Overdick M., Baumer C., Engel K.J., Fink J., Herrmann C., Kruger H., Simon M., Steadman R., Zeitler G. (2009). Status of direct conversion detectors for medical imaging with X-rays. IEEE Trans. Nucl. Sci..

[9] Strassburg M., Schroeter C., Hackenschmied P. (2011). CdTe/CZT under high flux irradiation. J. Instrum..

[10] Jahnke A., Matz R. (1999). Signal formation and decay in CdTe X-ray detectors under intense irradiation. Med. Phys..

[11] Szeles C., Soldner S.A., Vydrin S., Graves J., Bale D.S. (2007). Ultra high flux 2-D CdZnTe monolithic detector arrays for x-ray imaging applications. IEEE Trans. Nucl. Sci..

[12] Pekárek J., Dědič V., Franc J., Belas E., Rejhon M., Moravec P., Touš J., Voltr J. (2016). Infrared LED Enhanced Spectroscopic CdZnTe Detector Working under High Fluxes of X-rays. Sensors.

[13] Thomas B., Veale M.C., Wilson M.D., Seller P., Schneider A., Iniewski K. (2017). Characterisation of Redlen high-flux CdZnTe. J. Instrum..

[14] Vartsky D., Goldberg M., Eisen Y., Shamai Y., Dukhan R., Siffert P., Koebel J., Regal R., Gerber J. (1988). Radiation induced polarization in CdTe detectors. Nucl. Instrum. Methods Phys. Res. Sect. A Accel. Spectrom. Detect. Assoc. Equip..

[15] Bale D.S., Soldner S.A., Szeles C. (2008). A mechanism for dynamic lateral polarization in CdZnTe under high flux x-ray irradiation. Appl. Phys. Lett..

[16] Bale D.S., Szeles C. (2008). Nature of polarization in wide-bandgap semiconductor detectors under high-flux irradiation: Application to semi-insulating Cd1–xZnxTe. Phys. Rev. B.

[17] Bolotnikov A.E., Camarda G.S., Carini G.A., Cui Y., Li L., James R.B. (2016). Cumulative effects of Te precipitates in CdZnTe radiation detectors. Nucl. Instrum. Methods Phys. Res. Sect. A Accel. Spectrom. Detect. Assoc. Equip..

[18] Prokesch M., Soldner S.A., Sundaram A.G., Reed M.D., Li H., Eger J.F., Reiber J.L., Shanor C.L., Wray C.L., Emerick A.J. (2016). CdZnTe Detectors Operating at X-ray Fluxes of 100 Million. IEEE Trans. Nucl. Sci..

[19] Mehta R., Sharma B. (1973). Photoconductive gain greater than unity in CdSe films with Schottky barriers at the contacts. J. Appl. Phys..

[20] Soares S.F. (1992). Photoconductive gain in a Schottky barrier photodiode. Jpn. J. Appl. Phys..

[21] Wilson M.D., Barnes P., Cernik R.C., Hansson C.C., Jacques S., Jones L.L., Seller P., Sellin P.J., Sochi T., Veale M.C. Comparison of the X-ray performance of small pixel CdTe and CZT detectors. Proceedings of the IEEE Nuclear Science Symposuim & Medical Imaging Conference.

